# Toll-like receptor activation by helminths or helminth products to alleviate inflammatory bowel disease

**DOI:** 10.1186/1756-3305-4-186

**Published:** 2011-09-27

**Authors:** ShuMin Sun, XueLin Wang, XiuPing Wu, Ying Zhao, Feng Wang, XiaoLei Liu, YanXia Song, ZhiLiang Wu, MingYuan Liu

**Affiliations:** 1 Key Laboratory of Zoonosis Research, Ministry of Education, Institute of Zoonosis, Jilin University; Zoonosis Research Centre of State Key Laboratory for Molecular Virology and Genetic Engineering, Institute of Pathogen Biology, Chinese Academy of Medical Sciences, Changchun 130062, People's Republic of China; 2 Animal Science and Technology College, Inner Mongolia University for Nationalities, 028000 Tongliao, People's Republic of China; 3 Department of Parasitology, Gifu University Graduate School of Medicine, Yanagido 1-1, Gifu 501-1194, Japan

**Keywords:** Toll Like Receptors, Helminth, Inflammatory Bowel Disease

## Abstract

Helminth infection may modulate the expression of Toll like receptors (TLR) in dendritic cells (DCs) and modify the responsiveness of DCs to TLR ligands. This may regulate aberrant intestinal inflammation in humans with helminthes and may thus help alleviate inflammation associated with human inflammatory bowel disease (IBD). Epidemiological and experimental data provide further evidence that reducing helminth infections increases the incidence rate of such autoimmune diseases. Fine control of inflammation in the TLR pathway is highly desirable for effective host defense. Thus, the use of antagonists of TLR-signaling and agonists of their negative regulators from helminths or helminth products should be considered for the treatment of IBD.

## Background

Crohn' s disease (CD) and ulcerative colitis (UC) are two forms of inflammatory bowel disease (IBD) that are autoimmune-like disorders characterized by chronic, idiopathic inflammation of the intestinal mucosal tissue, which causes a range of symptoms including abdominal pain, severe diarrhoea, rectal bleeding and wasting [1,2]. Patients with UC and CD are at increased risk of developing colorectal cancer. Chronic inflammation is believed to promote carcinogenesis [3].

CD and UC are distinguished by the tissues affected: CD can affect any region of the gastrointestinal tract in a discontinuous and transmural manner, whereas pathology in UC is restricted to the surface mucosa of the colon, in particular the rectum [4]. Current treatment regimens, including anti-inflammatory and immunosuppressive agents, are not curative and only reduce the degree of intestinal inflammation associated with disease [5].

Genetic studies have provided new evidence to suggest that derangements in innate and adaptive immunity result in human IBD [2]. In 1989, the "hygiene hypothesis" was proposed by D.P. Strachan in an article that claimed an inverse relationship between the occurrence of hay fever and numbers of siblings [6]. According to the hypothesis, atopic disorders are due to reduced exposure to microorganisms in childhood [7]. IBD tends to emerge in childhood, occurs primarily in immunocompetent individuals and is most prevalent in westernized regions of the world [8]. Weinstock [9] proposed that the modern lifestyle lacking consistent exposure to intestinal helminths is an important environmental factor contributing to IBD. Cross-sectional studies on the relationship between skin prick tests and helminth infections suggested a general protective effect on the atopic reaction [10]. Nowadays, the concept is becoming more accepted, with accumulating evidence not only in atopic diseases but also in autoimmune inflammatory diseases [11]. Many studies have since demonstrated that helminth infections lower the risk of autoimmunity or allergy [12]. Thus, parasitic worms are important for shaping, or tuning, the development and the function of the immune systems of human beings. Helminths (nematodes, cestodes and trematodes) have been used in ameliorating chemically induced colitis in different models [13,14].

Khan *et al *supported these results by infecting mice with *Trichinella spiralis *and showed that mice were protected from colitis induced by an intrarectal challenge using dinitrobenzene sulfate (DNBS) [15]. Reardon *et al*. evidenced that mice infected with the tapeworm *Hymenolepis diminuta*, ameliorated dextran sodium sulfate (DSS)-induced colitis [16]. Helminths can attenuate experimentally induced IBD in animal models [17,18], but the work of Summers *et al*. also shows promise in that natural exposure to helminths, such as *T. suis*, affords protection from immunological diseases like CD [19,20]. Epidemiological and experimental data strongly support the hypothesis that a reduction in helminth infection is linked to a rise in the incidence rates of autoimmune diseases [21].

### Basic immunopathology of IBD

An important role for TLR signaling in the pathogenesis of IBD has been established through many studies over the last decade [22-24]. In the IBD-susceptible host, aberrant TLR signaling may contribute to destructive host responses and chronic inflammation, disturbing mucosal and commensal homeostasis and leading to many different clinical phenotypes [25]. Hyperactivation of the adaptive immune system, secondary to TLR deficiency, may drive tissue damage and progressive inflammation in IBD [26,27]. Characterization of different IBD-associated gene defects have highlighted fundamental, defining variability in TLR regulation and function, dependent on disease processes and predominant cell type involvement in the intestinal mucosa [28,29]. TLRs and pattern recognition receptors (PRRs) may be central to future progress in identifying novel approaches that may exploit innate immune functions as a means to prevent and/or treat IBD and related systemic manifestations.

It is now clear that the innate immune system comprised of TLRs and related molecules, plays a key role in the regulation of intestinal inflammation and in the recognition of invading pathogens [30]. TLRs comprise the major innate immune surveillance, recognition and response receptors central to efficient host defense and homeostasis of the intestinal mucosa [31,32]. There are currently 11 known mammalian TLRs. They are transmembrane receptors that are found either on the cell membrane (TLR1, 2, 4, 5 and 9) or on intracellular organelles (TLR3, 7 and 8) [33]. TLRs are expressed throughout the gastrointestinal (GI) tract on intestinal epithelial cells (IECs), myofibroblasts, enteroendocrine cells, and on immune cells within the lamina propria, such as T cells, and dendritic cells (DCs) [34-38]. Ligand binding to TLRs initiates signaling cascades that activate NF-κB, MAPK, and interferon response factors [39].

TLR molecules and their downstream signaling pathways play a crucial role in selected cell types in adaptive immunity and in activating innate immune cells of the immune system [40,41]. Given that this pathway is aberrantly expressed or activated in several diseases, it constitutes a potential target for therapeutic intervention. There is mounting evidence documenting that the interruption of this pathway at the level of TLR, myeloid differentiation factor-88 (MyD88), or IL-I receptor-associated kinase (IRAK) will improve therapeutic efficacy in autoimmunity and auto-inflammatory diseases [42-44]. On the contrary, the total abolition of these pathways may compromise the immune defense against invading infections and immune surveillance [45,46]. Actually, agonists of these pathways appear to be useful in IBD development. Hence, there is a need to mindfully select the therapeutic target in the TLR signaling cascade and closely regulate the degree of pathway activity so as to procure the ideal therapeutic end point [47].

**Figure 1:**
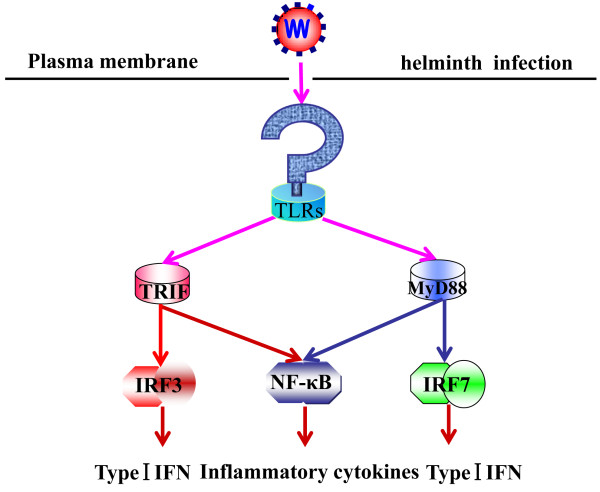
**TLR induction of inflammation by parasitic infection**. MyD88, TLRs and toll/IL-1 receptor domain-containing adaptor protein inducing interferon (TRIF) signal transduction is activated by helminth infection, which results in the activation of NF-κB, IRF7 and IRF3 for the induction of type I interferons (TRIF-dependent pathway). Activation of NF-κB is required for the induction of inflammatory cytokines.

In the intestine, the end result of TLR signaling is the activation of nuclear factor kappa-B (NF-κB), triggering off the induction of pro-inflammatory cytokines or interferon (IFN) response factors (IRFs), depending on the induction of type I interferons (Figure 1). TLR-dependent activation of NF-κB plays an important role in sustaining epithelial homeostasis as well as in regulating infections and inflammation, while the dysregulation of TLR-signaling is associated with the pathogenesis of IBD [48,49]. Recent findings on innate immunity-mediated regulation of intestinal pathophysiology prove that the development of new drugs targeting TLRs, including antagonists of TLR-signaling and agonists of their negative regulators, hold promise for new therapeutic strategies for intestinal inflammatory diseases [50].

### Helminth infection affects key aspects of gut inflammatory biology

Negative regulation of TLRs reduces pro-inflammatory cytokine production, protecting the host from autoimmune pathogenesis [51]. Helminths can both activate and negatively regulate TLRs, which suggests that the immune response to these infective helminths is under tight control [52]. Zhao *et al*. [53] reported that *Schistosoma japonicum *eggs could alleviate TNBS-induced colitis in mice. The mechanism for this action was assumed to be due to the regulation of T-helper cell 1/2 balance and TLR4 expression. In brief, these reports make a significant contribution in that helminths will execute positive therapy in IBD by targeting the TLR signaling pathway.

**Figure 2:**
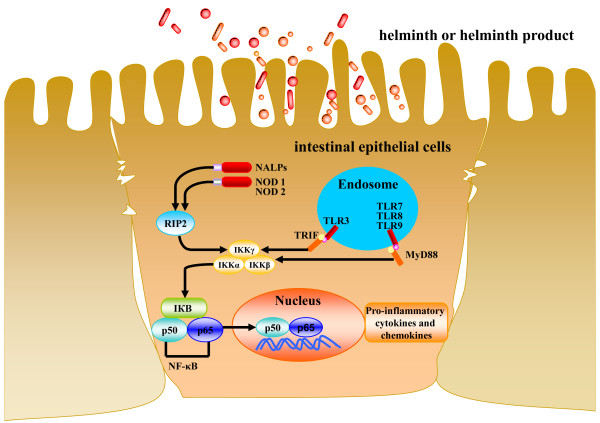
**Contribution of TLRs to mucous membrane immunity**. Pattern-recognition receptors, including toll-like receptors (TLRs) and nucleotide-binding oligomerization domain- (NOD)-like receptors (NLRs), are expressed by most IEC. TLR ligation leads to the recruitment of adaptor proteins, such as TIR domain-containing adaptor protein inducing interferon (TRIF), MyD88 (myeloid differentiation primary-response gene 88) and subsequent activation of several signaling modules, including mitogen-activated protein (MAP) kinase pathways NF-κB. Activation of PRRs by helminth infection advances a cascade of signaling events that results in the expression of pro-inflammatory cytokines and chemokines.

The critical roles of TLRs are to sustain the integrity of the epithelial barrier and to accelerate maturation of the mucosal immune system. Mice deficient in TLRs can develop intestinal inflammation [54]. IECs express TLRs that recognize specific molecular signatures of helminths, which can then trigger intracellular signaling pathways inducing the production of pro-inflammatory cytokines and chemokines (Figure 2). TLR responses are tightly regulated in order to induce protective responses while reducing excessive and detrimental inflammatory responses for IEC [55,56].

Recent studies have demonstrated that TLR signals can influence intestinal homeostasis [57]. One study proved that the expression levels of TLR-2, TLR-4, TLR-9 and TLR-11 were significantly raised in mouse IECs following infection with *Toxoplasma gondii *on day 8 post-infection [58]. Mucosal cells and consequent activation of signaling cascades including activator protein 1 (AP1), mitogen-activated protein kinases, NF-κB and IRFs can enhance the production of pro-inflammatory cytokines and antimicrobial peptides, as well as the maintenance of the epithelial barrier function and epithelial cell proliferation [59]. Hence, parasitic infection can maintain the epithelial barrier function and epithelial cell proliferation through TLR signaling pathways [60].

Intestinal parasitic infections also activate mucin hypersecretion, which is a key response of the innate immune system for intestinal homeostasis [61]. One study suggests that *Gymnophalloides seoi *antigen can induce mucin-related 2 (MUC2) expression by the activation of the TLR pathways in human IECs [62]. The expression and regulation of MUC genes were reported in rodents infected with intestinal nematodes, including *Trichinella spiralis *and *Nippostrongylus brasiliensis *[63]. These results suggest the possibility that the expression of the MUC2 gene may be closely associated with TLR pathways [64,65]. Consequently, helminthes, or their products, may promote the physical barrier function of IECs by TLR activation.

Thus, the fine control of inflammation by helminths in the TLR pathway is highly feasible for effective host defense via TLR-dependent pro-inflammatory cascades triggered by parasitic infections, which must be tightly regulated to avoid severe pathology or even mortality in IBD patients [51].

### Bioactive helminths or helminth products

**Figure 3:**
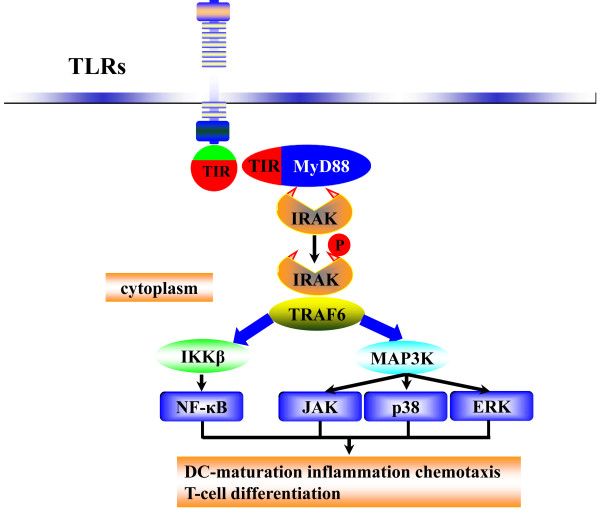
**TLR regulation of pro-inflammatory cytokines**. Activation of toll-like receptors and type I IL-1 receptors evoke inflammation in immune cells by sharing signaling cascades. TLRs expressed on professional immune cells (macrophages, dendritic, monocytes and microglia cells) discern and respond to helminth infection. TLRs are triggered by helminth or helminth products containing pathogen-associated molecular patterns (PAMPs). All TLR family members and the type I interleukin-1 receptor (IL-1RI) have specific intracellular TIR signaling domains. In response to activation by the corresponding ligands, TIR domains react with the TIR domains of the signaling adaptor MyD88, which convey the signal to a family of IL-1 receptor-associated kinases (IRAKs). Phosphorylation of IRAK, a serine-threonine kinase, by other IRAK family members provoke cascades of signaling through tumor necrosis factor receptor-associated factor 6 (TRAF6). TRAF6 relays a signal to I kappa B kinase (IKK) and to mitogen-activated protein kinase kinase (MAP3K). This signaling leads to transcriptional responses, mediated primarily by ERK, NF-κB and stress-activated protein kinases, for example JNK and p38, result in the expression of pro-inflammatory cytokines.

TLRs trigger an intracellular signaling cascade through the toll/IL-1 receptor (TIR) [66] and through the recruitment of adaptor molecules, such as TIR domain-containing adaptor molecule-1 (TICAM-1), MyD88 and TRIF, and TRIF-related adaptor molecule (TRAM) [67,68]. These adaptor molecules act independently, or in combination, based on the TLRs and trigger NF-κB, c-Jun-N-terminal kinase (JNK), mitogen-activated protein kinases (MAPK), p38, extracellular signal-regulated kinase (ERK) and NF-κB leading to the transcription of inflammatory and immunomodulatory genes including co-stimulatory molecules, cytokines and chemokines [69,70] (Figure 3). In IBD therapy by helminths or helminth products, negative regulation of TLR signaling is critical for the down regulation of gene activation in controlling overwhelming inflammation and pro-inflammatory cytokine production.

**Figure 4:**
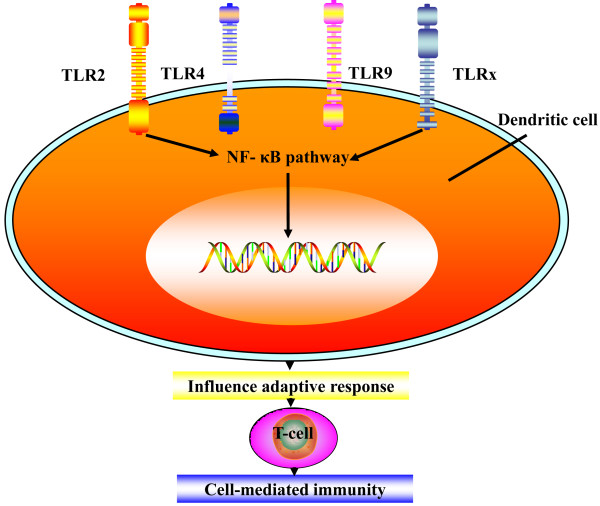
**Adaptive T-cell immune response induced by TLRS**. Mammalian toll-like receptors are expressed on all kinds of immune cells, including dendritic cells and monocytes. Activation of toll-like receptors induces signaling pathways that activate the transcription factor NF-B, leading to the transcription of genes that modulate and mediate immune responses. Activation of these pathways results in the release of pro-inflammatory cytokines, which affects the adaptive T-cell immune response.

A recent report indicates that helminth infection may alter TLR4 expression in mucosal T cells [37]. *Schistosoma *derived lysophosphatidyl-serine contains a helminth-specific acyl chain that, through influence on TLR2, promotes the differentiation of DCs that induce regulatory T cells, which secrete the anti-inflammatory cytokine interleukin-10 (IL-10) [71]. Studies conducted by Meyer *et al*. [72] suggest that the soluble fractions from *Schistosoma mansoni *eggs may alter TLR ligand-induced activation of DCs. The broad effect of excretory-secretory products (ESP) of *Fasciola hepatica *on different TLR signaling regulation could be an immediate action of these antigens (Ags) on TLR expression. Falcón demonstrated that ESP was also able to affect the MyD88-dependent signaling pathway [73]. These results indicate that different helminths may modulate the TLR expression of DCs and responsiveness of DCs to TLR ligands and finally stimulate cell-mediated immunity (Figure 4). Nevertheless, characterization of the signals induced by these immunomodulators suggest overturn of the normal TLR-induced MAPK and that NF-κΒ pathways lead to antigen presentation of an immature phenotype to antigen-presenting cells (APCs) that subsequently reduce levels of proinflammatory cytokines [74,75]. Logically, the biological characteristics of helminths should be considered for IBD therapy.

### Ameliorating the inflammation strategy

The intestinal tract is the largest and most complex immune environment in the human body. Successful therapy for these tissues will require accurate timing and targeting the optimal location. The number of therapeutics being developed for IBD has increased dramatically over the last 2 decades because of rapid gains in our understanding of the mechanisms of inflammation [76].

Disturbing TLR signaling by helminths or helminth products is expected to be a promising strategy in IBD treatment because TLR signaling can inhibit inflammatory responses in innate immune cells [77]. van Stijn *et al*. [78] demonstrate that TLR4 activation by worm glycolipids may elicit Th1 immune responses in *Schistosoma *infection. Donnelly *et al*. [79] showed that parasite proteases specifically degrade TLR3 within the endosome, which reduces macrophage activation in response to both TLR3 and TLR4 stimulation. Maintaining the epithelial barrier function and IEC proliferation by TLR signals[80] is another strategy of IBD therapy by the parasite or products derived from the parasite. In report of Lee *et al *[65], the intestinal trematode *G. seoi *was employed in inducing the expression of TLR4, TLR2 and the MUC2 gene in a human IEC. MUC2 has been used in alleviating ulcerative colitis of the IBD model mouse [81].

Cysteine proteases, excretory-secretory (ES) production and antigens from helminths with potential TLR ligands that may obtain more effective agonists or antagonists of a targeted function of TLRs signaling need to be considered in IBD treatment. One study showed that the major cysteine proteases secreted from *F. hepatica *and *S. mansoni *specifically disturb the MyD88-independent, TRIF-dependent signaling pathways of TLR4 and TLR3 for the modulation of the innate immune responses of their hosts [79]. These results clearly show the benefits of local treatment with helminth antigens for experimental colitis and prompt consideration of helminth antigen-based therapy for IBD, in lieu of infection with live parasites.

## Conclusions

Epidemiological, experimental and clinical data support the idea that helminths could provide protection against IBD. Correale and Farez [82] evidenced that a soluble egg Ag (SEA) obtained from *Schistosoma mansoni *exerts potent regulatory effects on both DCs and B cells through TLR2 regulation in patients with the autoimmune disease, multiple sclerosis. Summers *et al*. [19] demonstrated that it is safe to administer eggs from the porcine whipworm, *Trichuris suis*, to patients with CD and UC. The study suggests that it is possible to down-regulate aberrant intestinal inflammation in humans with helminthes. Local treatment using antagonists of TLR-signaling and agonists of their negative regulators from helminthes or helminth products ought to prompt consideration for treatment of IBD instead of infection with live parasites.

## List of abbreviations

IBD: inflammatory bowel disease; DCs: dendritic cells; Treg: regulatory T cell; TLR: Toll-like receptor; CD: Crohn's disease; UC: ulcerative colitis; DNBS: dinitrobenzene sulfate; DSS: dextran sodium sulfate; PRRs: pattern recognition receptors; GI: gastrointestinal; IECs: intestinal epithelial cells; MyD88: myeloid differentiation factor-88; IRAK: IL-I receptor-associated kinase; NF-κB: nuclear factor kappa-B; IFN: interferon; IRFs: interferon response factors; TRIF: toll/IL-1 receptor domain-containing adaptor protein inducing interferon; TIR: toll/IL-1 receptor; NOD: nucleotide-binding oligomerization domain; NLRs: nucleotide-binding oligomerization domain-like receptors; NALPs: neutrophilic alkaline phosphatases; RIP: regulated intramembrane proteolysis; MAP: mitogen-activated protein; AP1: activator protein 1; MUC2: mucin-related 2; TICAM-1: TIR domain-containing adapter molecule-1; TIRAP: TIR domain-containing adaptor protein; TRAM: TRIF-related adaptor molecule; JNK: c-Jun-N-terminal kinase; MAPK: mitogen-activated protein kinases; ERK: extracellular signal-regulated kinase; PAMPs: pathogen-associated molecular patterns; IL-1RI: type I interleukin-1 receptor; IRAKs: IL-1 receptor-associated kinases; TRAF6: tumor necrosis factor receptor-associated factor 6;. IKK: I kappa B kinase; ESP: excretory-secretory products; APCs: antigen-presenting cells; ES: excretory-secretory; SEA: soluble egg Ag.

## Competing interests

The authors declare that they have no competing interests.

## Authors' contributions

SMS, ZLW and MYL drafted the manuscript. XLW, XPW, YZ, FW, XLL and YXS collected material or generalized useful information from the collected material in the paper. All authors approved the final version of the manuscript.
